# Epidermal Growth Factor Stimulates Nuclear Factor-κB Activation and Heme Oxygenase-1 Expression via c-Src, NADPH Oxidase, PI3K, and Akt in Human Colon Cancer Cells

**DOI:** 10.1371/journal.pone.0104891

**Published:** 2014-08-14

**Authors:** Gi-Shih Lien, Ming-Shun Wu, Mauo-Ying Bien, Chien-Hsin Chen, Chien-Huang Lin, Bing-Chang Chen

**Affiliations:** 1 Division of Gastroenterology, Department of Internal Medicine, Wan Fang Hospital, Taipei Medical University, Taipei, Taiwan; 2 Graduate Institute of Clinical Medicine, College of Medicine, Taipei Medical University, Taipei, Taiwan; 3 School of Respiratory Therapy, College of Medicine, Taipei Medical University, Taipei, Taiwan; 4 Division of Pulmonary Medicine, Department of Internal Medicine, Taipei Medical University Hospital, Taipei, Taiwan; 5 Division of Colorectal Surgery, Department of Surgery, Wan Fang Hospital, Taipei Medical University, Taipei, Taiwan; 6 Graduate Institute of Medical Sciences, College of Medicine, Taipei Medical University, Taipei, Taiwan; Chang Gung University, Taiwan

## Abstract

Previous report showed that epidermal growth factor (EGF) promotes tumor progression. Several studies demonstrated that growth factors can induce heme oxygenase (HO)-1 expression, protect against cellular injury and cancer cell proliferation. In this study, we investigated the involvement of the c-Src, NADPH oxidase, reactive oxygen species (ROS), PI3K/Akt, and NF-κB signaling pathways in EGF-induced HO-1 expression in human HT-29 colon cancer cells. Treatment of HT-29 cells with EGF caused HO-1 to be expressed in concentration- and time-dependent manners. Treatment of HT-29 cells with AG1478 (an EGF receptor (EGFR) inhibitor), small interfering RNA of EGFR (EGFR siRNA), a dominant negative mutant of c-Src (c-Src DN), DPI (an NADPH oxidase inhibitor), glutathione (an ROS inhibitor), LY294002 (a PI3K inhibitor), and an Akt DN inhibited EGF-induced HO-1 expression. Stimulation of cells with EGF caused an increase in c-Src phosphorylation at Tyr406 in a time-dependent manner. Treatment of HT-29 cells with EGF induced an increase in p47*^phox^* translocation from the cytosol to membranes. The EGF-induced ROS production was inhibited by DPI. Stimulation of cells with EGF resulted in an increase in Akt phosphorylation at Ser473, which was inhibited by c-Src DN, DPI, and LY 294002. Moreover, treatment of HT-29 cells with a dominant negative mutant of IκB (IκBαM) inhibited EGF-induced HO-1 expression. Stimulation of cells with EGF induced p65 translocation from the cytosol to nuclei. Treatment of HT-29 cells with EGF induced an increase in κB-luciferase activity, which was inhibited by a c-Src DN, LY 294002, and an Akt DN. Furthermore, EGF-induced colon cancer cell proliferation was inhibited by Sn(IV)protoporphyrin-IX (snPP, an HO-1 inhibitor). Taken together, these results suggest that the c-Src, NADPH oxidase, PI3K, and Akt signaling pathways play important roles in EGF-induced NF-κB activation and HO-1 expression in HT-29 cells. Moreover, overexpression of HO-1 mediates EGF-induced colon cancer cell proliferation.

## Introduction

Approximately one million cases of colon cancer are diagnosed worldwide each year, and an increasing trend in the incidence of colon cancer in Asian countries was reported in recent years [1]. Previous reports indicated that the intake of red and processed meats is associated with an increased risk of colorectal cancer because red meat contains approximately 10-fold higher levels of heme than white meat [2]. Heme oxygenase (HO) plays vital roles in physiological iron homeostasis, antioxidant defense, and cancer cell proliferation [3]. HO catalyzes the conversion of heme to biliverdin, releasing equimolar amounts of carbon monoxide, and concomitant induction of iron-sequestering ferritin [4]. Three isoforms of HO (HO-1, -2, and -3) were identified [5]. HO-1 is an inducible enzyme caused by growth factors including transforming growth factor (TGF)-β and epidermal growth factor (EGF), reflecting the main role of this enzyme in protecting against oxidative injury [6], [7]. Moreover, HO-1 is often highly upregulated in colon cancer compared to surrounding normal tissue, suggesting that cancer cells highly expressing HO enjoy a growth advantage and provide cellular resistance against reactive oxygen species (ROS)-mediated anticancer therapies [8]–[10].

The importance of EGF in the development of colon cancer was emphasized in recent years [11]. A growing body of evidence suggests that EGF regulates multiple biological functions such as cancer cell progression, cell proliferation, and metastasis [11]. The EGF receptor (EGFR) was shown to participate in colon cancer development [11]. EGF binds to the extracellular domain of the EGFR which activates downstream signaling pathways including the c-Src and phosphatidyl inositol 3-kinase (PI3K)/Akt pathways [12], [13]. A previous report indicated that overexpression of HO-1 plays a protective role in attenuating cellular damage and cancer cell survival [6], [7]. However, little is known about how EGF regulates the induction of HO-1 protein expression.

Expression of the *HO-1* gene is primarily regulated at the transcription level by activating transcription factors including nuclear factor (NF)-κB, activating protein (AP)-2, and the heat shock-responsive element (HSE) [14], [15]. NF-κB is an important transcription factor for regulating HO-1 expression [16]. At rest, NF-κB binding to IκBα prevents NF-κB nuclear translocation and transcription activity [17]. However, growth factors induce IκB kinase (IKK) activation, IκBα phosphorylation, and IκBα degradation. This process releases active NF-κB, which is then translocated from the cytosol to nuclei, to bind the HO-1 promoter region and induce *HO-1* gene expression [16], [18]. Several reports showed that EGF-induced NF-κB activation occurs through multiple EGFR-dependent signaling molecules, including PI3K, protein kinase C (PKC), and IKK signaling pathways [19]. Our previous study revealed that TGF-β induced HO-1 expression via the PI3K/Akt-dependent NF-κB signaling pathway [6]. However, little is known about the signal transduction event; in particular, the c-Src, NADPH oxidase, ROS, and PI3K/Akt pathways, which lead to activation of NF-κB and the expression of HO-1 by EGF stimulation, are not well described.

Several studies demonstrated that c-Src and NADPH oxidase play important roles in inducing gene expressions [20], [21]. A previous report demonstrated that thrombin induced HO-1 expression and was dependent on c-Src-mediated NF-κB activation [20]. It was recently discovered that NADPH oxidase generation of ROS production is a defensive response by a host to apoptosis and cell transformation [22]. NADPH oxidase is regulated by p47*^phox^* which is capable of supporting activation of NADPH oxidase [23]. It known that EGF stimulates NADPH oxidase activity to produce superoxide, and the generated superoxide is rapidly dismutated to H_2_O_2_, leading to EGF-induced physiological responses [24]. However, little information is available about the role of NADPH oxidase in regulating NF-κB activation and HO-1 expression following EGF stimulation in human colon cancer cells. Our findings revealed that EGF triggering of the c-Src, NADPH oxidase, ROS, and PI3K/Akt signaling pathways leading to activation of NF-κB plays an important role in EGF-induced HO-1 expression in human lung colon cancer cells. Moreover, HO-1 is involved in EGF-induced colon cancer cell proliferation.

## Materials and Methods

### Materials

EGF was obtained from PeproTech (London, UK). LY 294002, diphenyleneiodonium chloride (DPI), glutathione, and 2′,7′-dichloroflurorescein diacetate (DCF-DA) were purchased from Sigma (St. Louis, MO, USA). A dominant negative mutant (DN) of IκBα (IκBαM) was purchased from Clontech (Mountain View, CA, USA). pGL2-ELAM-Luc (which is under the control of one NF-κB binding site) and pBK-CMV-Lac Z were kindly provided by Dr. Wan-Wan Lin (National Taiwan University, Taipei, Taiwan). A DN of Akt (Akt DN) was kindly provided by Dr. Che-Ming Teng (National Taiwan University, Taipei, Taiwan). The pcDNA plasmid was provided by Dr. M.-C. Chen (Taipei Medical University, Taipei, Taiwan). A c-Src DN was purchased from Upstate Biotechnology (Lake Placid, NY, USA). Fetal calf serum (FCS), penicillin/streptomycin, and Lipofectamine Plus reagent were purchased from Life Technologies (Gaithersburg, MD, USA). Sn(IV)protoporphyrin-IX (snPP) was purchased from Frontier Scientific (Logan, UT, USA). Control small interfering (si)RNA, EGFR siRNA, and antibodies specific for HO-1, c-Src, p47*^phox^*, Akt1/2, EGFR, Na^+^/K^+^ ATPase, p65, IκBα, and anti-mouse and anti-rabbit immunoglobulin G (IgG)-conjugated horseradish peroxidases (HRPs) were purchased from Santa Cruz Biotechnology (Dallas, TX, USA). Akt phosphorylated at Ser473 and c-Src phosphorylated at Tyr416 were purchased from New England Biolabs (Beverly, MA, USA). Lamin A/C was purchased from GeneTex (Ipswich, CA, USA). AG1478 and BrdU cell proliferation assay kit were purchased from Merck (Darmstadt, Germany). All materials for sodium dodecylsulfate polyacrylamide gel electrophoresis (SDS-PAGE) were purchased from Bio-Rad (Hercules, CA, USA).

### Cell culture

HT29 human colon cancer cells were obtained from the American Type Culture Collection (Livingstone, MT, USA), and cells were maintained in RPMI 1640 containing 10% FCS, 100 U/ml penicillin G, and 100 µg/ml streptomycin in a humidified 37°C incubator. After reaching confluence, cells were seeded onto 6-cm dishes for Western blotting and a reverse transcription polymerase chain reaction (RT-PCR), onto 12-well plates for cell transfection and the κB-luciferase activity assay, and onto 96-well plates for the BrdU cell proliferation and ROS generation assays.

### Western blot analysis

To determine expressions of HO-1, c-Src phosphorylated at Tyr416, Akt phosphorylated at Ser473, c-Src, Akt1/2, and EGFR in HT-29 cells, proteins were extracted, and a Western blot analysis was performed as described previously [6]. Briefly, HT-29 cells were cultured in 6-cm dishes. After reaching confluence, the growth medium was removed and replaced with 2 ml of RPMI 1640 without FCS for 24 h. Cells were treated with the vehicle and EGF, or pretreated with specific inhibitors as indicated followed by EGF. After incubation, cells were washed twice in ice-cold phosphate-buffered saline (PBS) and solubilized in lysis buffer containing 10 mM Tris (pH 7.0), 140 mM NaCl, 2 mM phenylmethylsulfonyl fluoride (PMSF), 5 mM dithiothreitol, 0.5% NP-40, 0.05 mM pepstatin A, and 0.2 mM leupeptin. Samples of equal amounts of protein (50 µg) were subjected to SDS-PAGE, then transferred onto polyvinylidene fluoride (PVDF) membrane which were then incubated in Tris-buffered saline with 0.1% Tween-20 (TBST) buffer containing 5% bovine serum albumin. Proteins were visualized by specific primary antibodies and then incubated with HRP-conjugated secondary antibodies. The immunoreactivity was detected using enhanced chemiluminescence following the manufacturer’s instructions. Quantitative data were obtained using a computing densitometer with scientific imaging systems (Eastman Kodak, Rochester, NY, USA).

### RNA extraction and the RT-PCR

To amplify human colon cancer cell HO-1 mRNA, specific primers were synthesized. The HO-1 primers used were: sense 5′-CTG TGT AAC CTC TGC TGT TCC-3′ and antisense 5′-CCA CAC TACCTG AGT CTA CC-3′. β-actin mRNA levels were used as an internal control. The β-actin primers used were: sense 5′-GAC TAC CTC AAG ATC CT-3′ and antisense 5′-CCA CAT CTG CTG GAA GGT GG-3′. HT-29 cells were seeded onto 6-cm dishes. After reaching conference, the medium was aspirated and replaced with basal medium devoid of FBS overnight, after which cells were stimulated with EGF for different time intervals. Total RNA was purified using the TRI REAGENT (Molecular Research Center, Cincinnati, OH, USA), and an RT-PCR carried out using an RT-PCR kit (Epicentre, Madison, WI, USA), according to the manufacturer’s instructions, using 10 µl of total RNA as a template. Equal amounts (10 µg of cDNA) of each PCR product were PCR-amplified with Tag polymerase in 35 cycles consisting of 30 s at 95°C, 30 s at 58°C, and 1 min at 72°C. The amplified cDNA was run on 2% agarose gels and visualized with ethidium bromide. The RT samples were also used to generate β-actin PCR products and their amount was used as an internal control.

### Transfection and the κB-luciferase assay

HT-29 cells (2×10^5^) were seeded onto 12-well plates, and cells were transfected the following day using Lipofectamine Plus reagent containing 0.5 µg of pGL2-ELAM-Luc and 0.5 µg of pBK-CMV-Lac Z. After 24 h, the medium was aspirated and replaced with fresh RPMI 1640 devoid of FCS, and then cells were stimulated with EGF (0.3∼10 ng/ml) for another 24 h before being harvested. To assess the effects of the indicated inhibitors, drugs were added to cells 20 min before the addition of EGF. To assess the effects of the c-Src DN and Akt DN, cells were cotransfected with pGL2-ELAM-Luc and pBK-CMV-Lac Z. Luciferase activity was determined with a luciferase assay system (Promega, Madison, WI, USA), and was normalized on the basis of Lac Z expression. The level of induction of luciferase activity was compared as a ratio of cells with and without stimulation.

### Analysis of p47*^phox^* translocation

To detect p47*^phox^* translocation, cytosolic and membrane fractions were separated as described previously [25]. Briefly, HT-29 cells were treated with EGF for the indicated concentrations or for the various time intervals. After incubation, cells were placed on ice, rinsed with PBS, resuspended in homogenization buffer (20 mM Tris-HCl, 0.5 mM EGTA, 2 mM EDTA, 2 mM DTT, 0.5 mM PMSF, and 10 µg/ml leupeptin (pH 7.5)) and sonicated. The lysate was separated into cytosolic and membrane fractions by centrifugation at 40,000×*g* for 45 min. Levels of the p47*^phox^* protein in the cytosolic and membrane fractions were determined by a Western blot analysis. α-tubulin and Na^+^/K^+^ ATPase were respectively used as the internal controls of the cytosolic and membrane fractions.

### Analysis of p65 translocation

To detect p65 translocation, HT-29 cells were treated with EGF for the indicated concentrations or for the various time intervals. The cytosolic and nuclear protein fractions were then separated as described previously [25]. Briefly, HT-29 cells were washed with ice-cold PBS, and pelleted. Cell pellets were resuspended in hypotonic buffer (10 mM HEPES (pH 7.9), 10 mM KCl, 0.5 mM DTT, 10 mM aprotinin, 10 mM leupeptin, and 20 mM PMSF) for 15 min on ice, and vortexed for 10 s. Nuclei were pelleted by centrifugation at 15,000×*g* for 1 min. Supernatants containing cytosolic proteins were collected. A pellet containing nuclei was resuspended in hypertonic buffer (20 mM HEPES (pH 7.6), 25% glycerol, 1.5 mM MgCl_2_, 4 mM EDTA, 0.05 mM DTT, 10 mM aprotinin, 10 mM leupeptin, and 20 mM PMSF) for 30 min on ice. Supernatants containing nuclear proteins were collected by centrifugation at 15,000×*g* for 2 min. Protein levels of p65 in the cytosolic and nuclear fractions were determined by Western blot analysis. α-tubulin and lamin A/C were respectively used as the cytosol and nuclear internal controls.

### Determination of ROS production

ROS were determined as described previously [26]. Briefly, HT-29 cells were seeded onto 96-well plates in RPMI 1640 containing 10% FCS overnight. The next day, the medium was aspirated and replaced with fresh RPMI 1640 devoid of FCS. After 24 h, cells were treated with the ROS-sensitive DCF for 15 min, and then stimulated with EGF for indicated concentrations or for various time intervals. To assay the effect of DPI (10 µM), the drug was added to cells 20 min before the addition of EGF. The fluorescence was determined with a Varioskan Flash fluorescence plate reader (Thermo Electron Corporation, Marietta, OH, USA) with excitation at 485 nm and emission at 528 nm.

### BrdU cell proliferation assay

HT-29 cells (7.5×10^3^ cells/well) were seeded onto 96-well plates in RPMI 1640 containing 10% FCS. The next day, the medium was aspirated and replaced with fresh RPMI 1640 devoid of FCS overnight. Cells were pretreated with snPP (3 µM) for 20 min, and then stimulated with EGF (10 ng/ml) for another 48 h. BrdU was added to cells during the last 2 h of incubation. After removing the labeling medium, cells were fixed and DNA was denaturized. The incorporated BrdU was labeled by a monoclonal anti-BrdU antibody and a goat anti-mouse antibody conjugated with peroxidase. Immune complexes were detected by the subsequent substrate reaction and quantified by measuring the absorbance at 450 nm using a microplate reader.

### Statistical analysis

Results are presented as the mean ± standard error of the mean (SEM) from at least three independent experiments. A one-way analysis of variance (ANOVA) followed by, when appropriate, Dunnett’s multiple-comparisons test was used to determine the statistical significance of the difference between means. A *p* value of < 0.05 was considered statistically significant.

## Results

### EGF induces HO-1 expression in HT-29 cells

**Figure 1:**
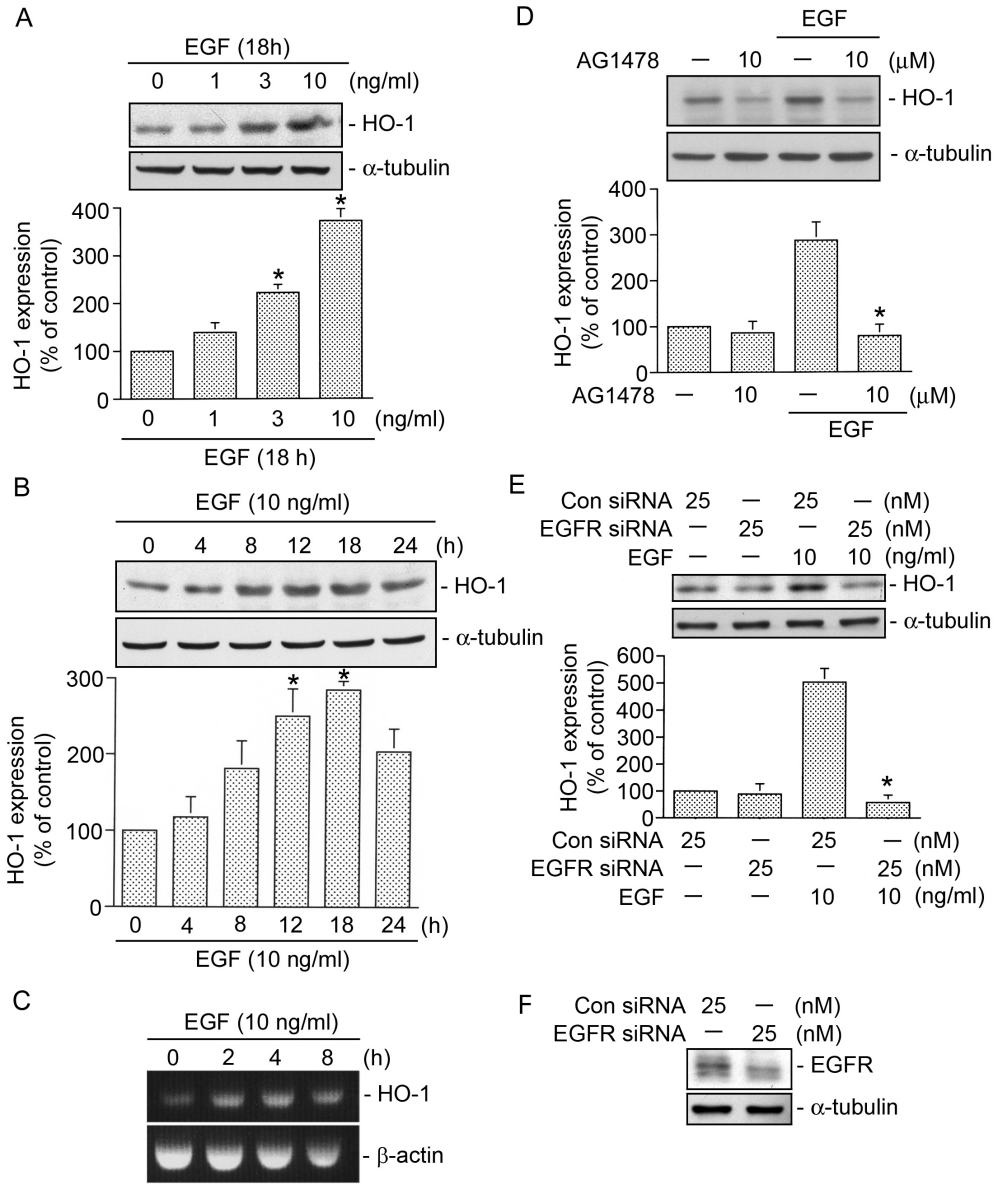
Epidermal growth factor (EGF) induces heme oxygenase (HO)-1 expression. A, HT-29 cells were incubated with various concentrations of EGF for 18 h, and then HO-1 and α-tubulin protein levels were determined. Immunoblots are representative of three experiments, which are presented as the mean ± SEM. *p<0.05, compared to the control group. B, Cells were incubated for various time intervals with EGF (10 ng/ml), and then HO-1 and α-tubulin protein levels were determined. Immunoblots are representative of three experiments, which are presented as the mean ± SEM. *p<0.05, compared to the control group. C, Cells were treated for various time intervals with EGF (10 ng/ml). Total RNA was prepared, and a RT-PCR was carried out as described in “Materials and Methods”. Taces represent results from three independent experiments. D, Cells were pretreated for 30 min with 10 µM AG1478 and then stimulated with 10 ng/ml EGF for another 18 h. After incubation, HO-1 and α-tubulin protein levels were determined. Immunoblots are representative of three experiments, which are presented as the mean ± SEM. *p<0.05, compared to EGF treatment. E, Cells were transiently transfected with 25 nM of control siRNA (con siRNA) or 25 nM of EGFR siRNA for 24 h and then stimulated with EGF (10 ng/ml) for another 18 h. Cells lysates were prepared and then immunoblotted with antibodies for HO-1 or α-tubulin. Immunoblots are representative of three experiments, which are presented as the mean ± S.E.M. *p<0.05, compared to EGF treatment. F, Cells were transiently transfected with 25 nM of control siRNA (con siRNA) or 25 nM of EGFR siRNA for 24 h. Whole-cell lysates were prepared and then immunoblotted with antibodies for the EGFR or α-tubulin. Traces represent results from three independent experiments.

Many studies revealed that HO-1 expression play an important in protecting aganist cancer cell death. Human HT-29 colon cancer cells were chosen to investigate the signal pathways of EGF in HO-1 expression. Treatment with EGF (1∼10 ng/ml) for 18 h induced HO-1 protein expression in a concentration-dependent manner (Fig. 1A); this induction also occurred in a time-dependent manner, beginning at 8 h and reaching a maximum at 12∼18 h (Fig. 1B). After 18 h of treatment with 10 ng/ml EGF, the HO-1 protein had increased by 185±11% (Fig. 1B). Next, we determined whether EGF can induce HO-1 mRNA expression. After treatment, induction of HO-1 mRNA had begun at 2 h and reached a maximum at 4 h after EGF (10 ng/ml) treatment (Fig. 1C). As previously mentioned, the EGFR is necessary for EGF responses. To examine whether the EGFR is involved in EGF-induced HO-1 expression, AG1478 was used. Figure 1D shows that pretreatment of HT-29 cells with AG4178 (10 µM) completely inhibited EGF-induced HO-1 expression (*n = *3). To further confirm the role of the EGFR in EGF-induced HO-1 expression, EGFR siRNA was used. As shown in Fig. 1E, transfection with EGFR siRNA (25 nM) also completely inhibited EGF-induced HO-1 expression (*n* = 3) (Fig. 1E). To confirm results of EGFR siRNA experiment, we also used EGFR siRNA to suppress EGFR protein expression in HT-29 cells. We found that that EGFR siRNA markedly inhibited EGFR protein expression (Fig. 1F). These results suggest that EGFR is involved in EGF-induced HO-1 expression in HT-29 cells.

### c-Src is involved in EGF-induced HO-1 expression in HT-29 cells

**Figure 2:**
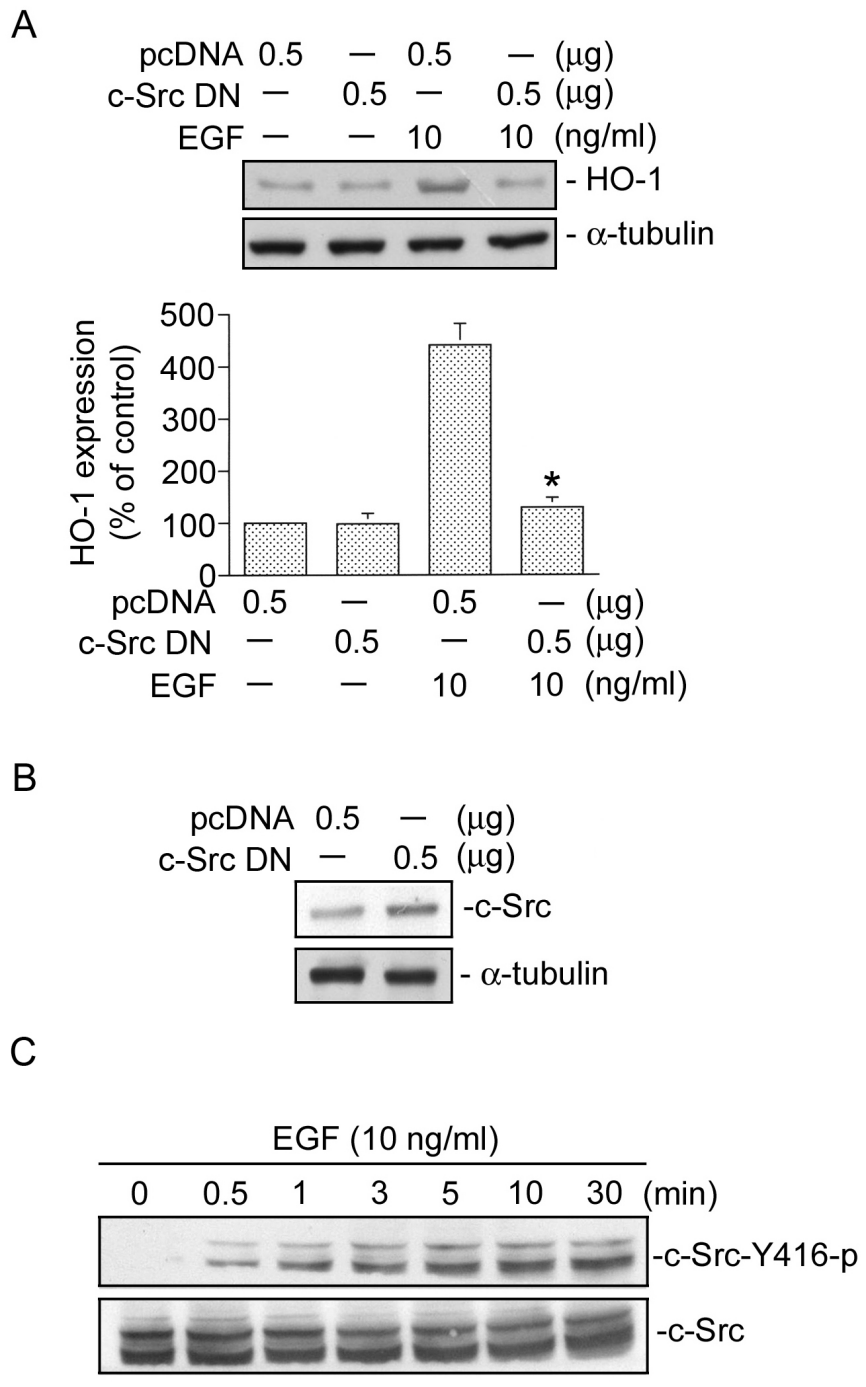
c-Src is involved in epidermal growth factor (EGF)-induced heme oxygenase (HO)-1 expression in HT-29 cells. A, HT-29 cells were transiently transfected with 0.5 µg of pcDNA or 0.5 µg of a dominant negative mutant of c-Src (c-Src DN) for 24 h. Cells were treated with EGF (10 ng/ml) for another 18 h. After incubation, HO-1 and α-tubulin protein levels were determined. Immunoblots are representative of three experiments, which are presented as the mean ± SEM. *p<0.05, compared to EGF treatment. B, Cells were transiently transfected with either 0.5 µg of pcDNA or 0.5 µg of c-Src DN for 24 h. Levels of c-Src or α-tubulin protein expressions were determined by a Western blot analysis. Traces represent results from three independent experiments. C, HT-29 cells were incubated with 10 ng/ml EGF for 0∼30 min. Cell lysates were prepared, and c-Src Tyr416 phosphorylation was determined by immunoblotting using a phospho-c-Src Tyr416 antibody. Immunoblots are representative of three experiments with similar results.

To examine whether c-Src, a downstream protein of the EGFR [12], might play a crucial role in EGF-induced HO-1 expression, a c-Src DN plasmid was used. As shown in Fig. 2A, transfection of HT-29 cells with the c-Src DN (0.5 µg) inhibited the EGF-induced increase in HO-1 expression by 91±6% (*n* = 3). Moreover, the level of c-Src protein was highly expressed in c-Src DN plasmid-transfected HT-29 cells compared to pcDNA plasmid-transfected HT-29 cells (Fig. 2B). Regulation of c-Src activation occurs as a result of the phosphorylation of multiple sites on specific residues, including Tyr416 [25]. Next, we further examined c-Src phosphorylation at Tyr416 by EGF stimulation in HT-29 cells using the anti-phospho-c-Src antibody at Tyr416. Figure 2C shows that treatment of HT-29 cells with 10 ng/ml EGF induced an increase in phosphorylation of c-Src at Tyr416 in a time-dependent manner. Phosphorylation of c-Src at Tyr416 began at 0.5 min and was sustained until 30 min after EGF stimulation (Fig. 2C, top panel). The protein level of c-Src was not affected by EGF stimulation (Fig. 2C, bottom panel). These results suggest that c-Src activation is required for EGF-induced HO-1 expression.

### Involvement of NADPH oxidase and ROS in EGF-induced HO-1 expression in HT-29 cells

**Figure 3:**
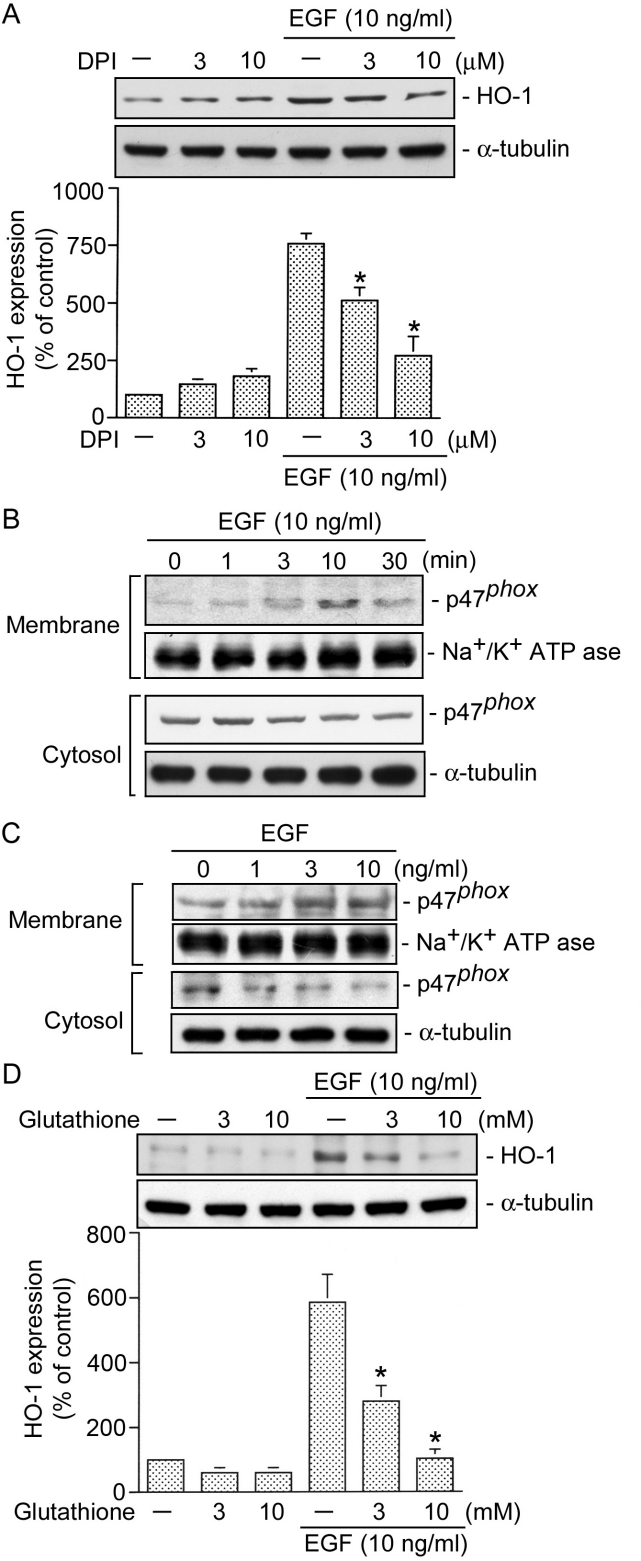
NADPH oxidase and reactive oxygen species (ROS) are involved in epidermal growth factor (EGF)-induced heme oxygenase (HO)-1 expression in HT-29 cells. HT-29 cells were pretreated for 30 min with 3∼10 µM DPI (A) and 3∼10 mM glutathione (D) and then stimulated with 10 ng/ml EGF. After an 18h incubation, HO-1 and α-tubulin protein levels were determined. Immunoblots are representative of three experiments, which are presented as the mean ± SEM. *p<0.05, compared to EGF treatment. HT-29 cells were treated with 10 ng/ml EGF for the indicated time intervals (B) or treated with different concentrations of EGF (C). The cytosolic and membrane fractions were then isolated, and protein levels of p47*^phox^* in the cytosolic and membrane fractions were determined by a Western blot analysis. Na^+^/K^+^ ATPase and α-tubulin were respectively used as the membrane and cytosolic internal controls. Typical traces represent three experiments with similar results.

c-Src might activate a number of signal pathways, including NADPH oxidase [27]. A previous study demonstrated that HT-29 cells predominantly expressed NADPH oxidase 1 [28]. To determine whether NADPH oxidase plays a crucial role in EGF-induced HO-1 expression, the NADPH oxidase inhibitor, DPI [29], was used. Figure 3A shows that EGF-induced HO-1 expression was inhibited by DPI (3 and 10 µM) in a concentration-dependent manner. When HT-29 cells were treated with 10 µM DPI, EGF-induced HO-1 expression was inhibited by 86±13% (*n = *3) (Fig. 3A). A previous study demonstrated that induction of p47*^phox^* translocation from the cytosol to membranes resulted in an increase in NADPH oxidase activity [27]. We next attempted to determine whether EGF activates NADPH oxidase by examining the translocation of p47*^phox^* from the cytosol to the membrane fraction using Western blot analysis. Stimulation of cells with 10 ng/ml EGF for 0∼30 min resulted in translocation of p47*^phox^* from the cytosolic fraction to the membrane fraction beginning at 3 min, the effect was sustained to 10 min, and declined by 30 min (Fig. 3B). Moreover, we found that incubation of cells with EGF (1∼10 ng/ml) produced a concentration-dependent increases in the translocation of p47*^phox^* from the cytosolic fraction to the membrane fraction (Fig. 3C). A previous study suggested that NADPH oxidase-generated ROS production participates in the signaling pathway that leads to the induction of HO-1 expression by treatment with a cigarette smoke extract [29]. To examine whether ROS might mediate EGF-induced HO-1 expression, glutathione was used. As shown in Fig. 3D, treatment of cells with glutathione (3∼10 mM) markedly inhibited EGF-induced HO-1 expression. When cells were treated with 10 mM glutathione, EGF-induced HO-1 expression was attenuated by 91±6% (*n* = 3). (Fig. 3D). These results suggest that NADPH oxidase and ROS are involved in EGF-induced HO-1 expression in human colon cancer cells.

### NADPH oxidase is involved in EGF-induced ROS production in HT-29 cells

**Figure 4:**
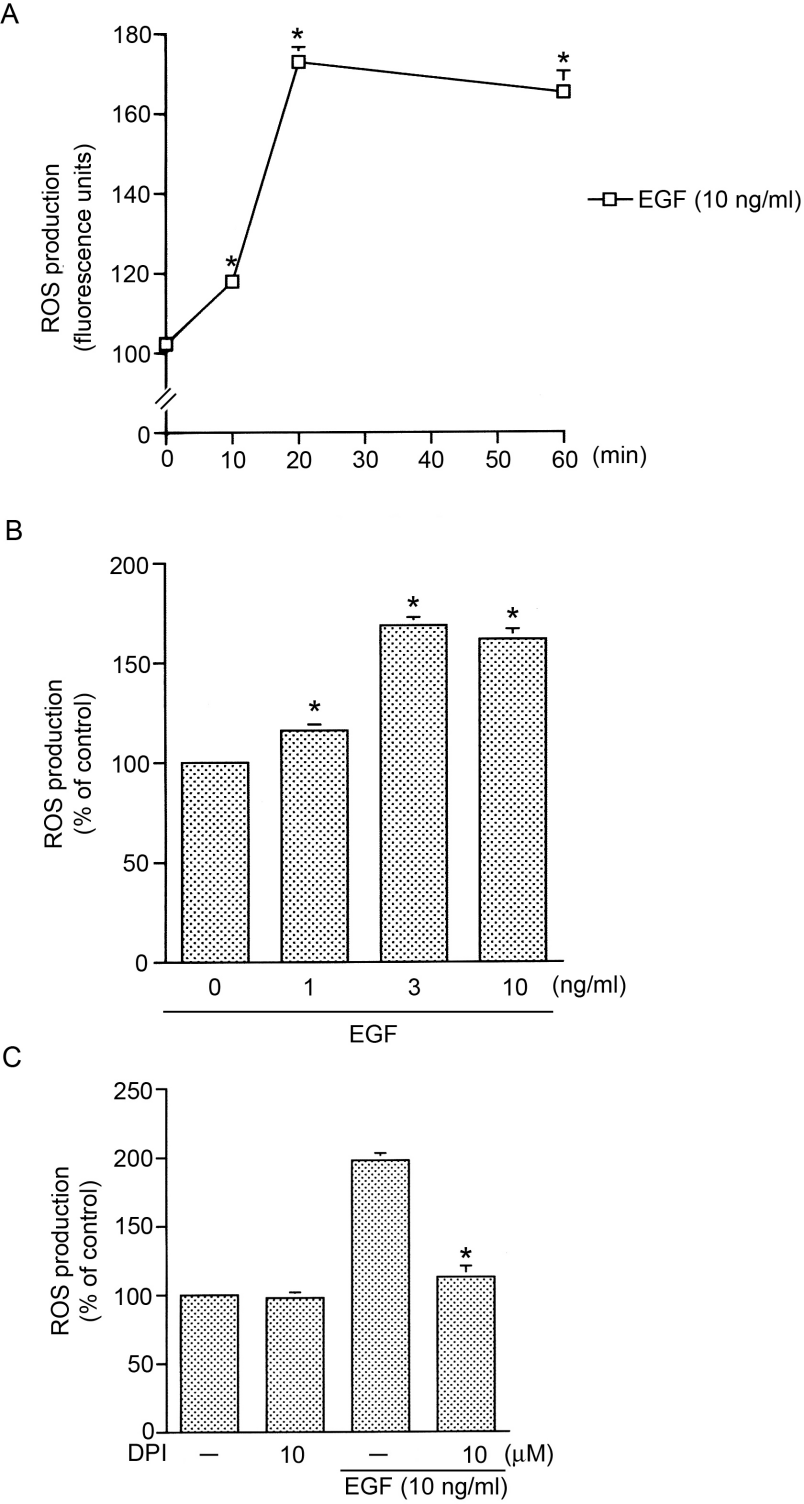
NADPH oxidase is involved in epidermal growth factor (EGF)-induced reactive oxygen species (ROS) production in HT-29 cells. HT-29 cells were incubated for various time intervals with EGF (10 ng/ml) (A) or incubated with EGF (1∼10 ng/ml) for 20 min, and then ROS production was determined. Data are representative of three experiments, which are presented as the mean ± S.E.M. *p<0.05, compared to the control group. C, HT-29 cells were pretreated for 30 min with 10 µM DPI and then stimulated with 10 ng/ml EGF. After 20 min of incubation, ROS production was determined. Data are representative of three experiments, which are presented as the mean ± S.E.M. *p<0.05, compared to EGF treatment.

A previous report demonstrated that EGF induced an increase in ROS generation in HT-29 cells [30]. Next, we investigated the role of NADPH oxidase in EGF-induced ROS production. Figure 4A and 4B show that treatment of HT-29 cells with EGF induced an increase in ROS generation in time- and concentration-dependent manners (Fig. 4A, 4B). After 20 min of treatment with 10 ng/ml EGF, the ROS production had increased by 62±5% (*n* = 3) (Fig. 4B). Moreover, pretreatment of cells with DPI (10 µM) markedly inhibited EGF-induced ROS generation by 85±8% (*n* = 3) (Fig. 4C). These results suggest that NADPH oxidase mediates EGF-induced ROS production in HT-29 cells.

### PI3K/Akt is involved in EGF-induced HO-1 expression in HT-29 cells

**Figure 5:**
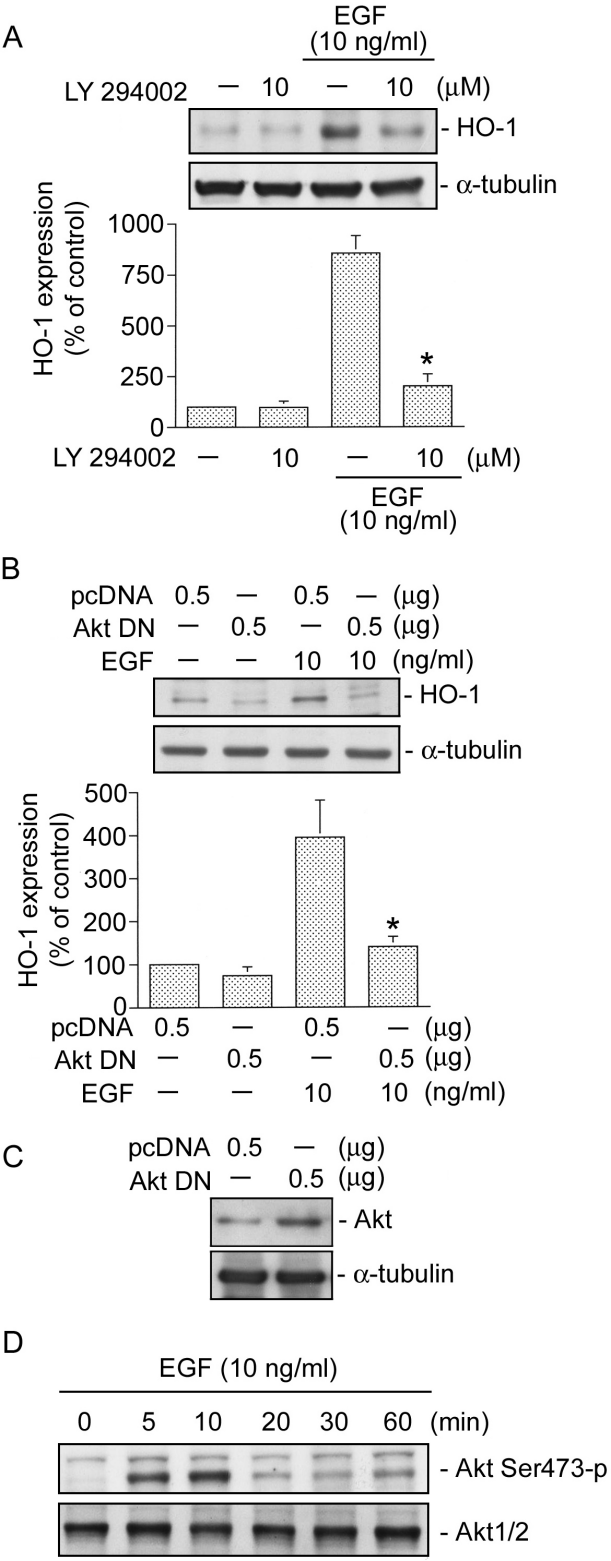
PI3K/Akt is involved in epidermal growth factor (EGF)-induced heme oxygenase (HO)-1 expression in HT-29 cells. HT-29 cells were pretreated for 30 min with 10 µM LY 294002 (A) or were transiently transfected with 0.5 µg of pcDNA or 0.5 µg of a dominant negative mutant of Akt (Akt DN) (B) for 24 h. Cells were then stimulated with EGF (10 ng/ml) for another 18 h. After incubation, HO-1 and α-tubulin protein levels were determined. Immunoblots are representative of three experiments, which are presented as the mean ± SEM. *p<0.05, compared to EGF treatment. C, Cells were transiently transfected with either 0.5 µg of pcDNA or 0.5 µg of the Akt DN for 24 h. Levels of Akt or α-tubulin protein expressions were determined by a Western blot analysis. Traces represent results from three independent experiments. D, HT-29 cells were incubated with 10 ng/ml EGF for 0∼60 min. Cell lysates were prepared, and Akt Ser473 phosphorylation was determined by immunoblotting using the phospho-Akt Ser473 antibody. Immunoblots are representative of three experiments with similar results.

A previous study demonstrated that PI3K/Akt plays an important role in HO-1 expression [31]. To understand the connection between HO-1 expression of EGF and its PI3K/Akt signaling pathway, the PI3K inhibitor (LY 294002) and an Akt DN, were used. As shown in Figure 5A, EGF-induced HO-1 expression was inhibited by 10 µM LY 294002 by 85±8% (Fig. 5A). Moreover, transfection of HT-29 cells with 0.5 µg of the Akt DN also inhibited EGF-induced HO-1 expression by 78±8% (Fig. 5B). Moreover, the level of Akt protein was highly expressed in Akt DN plasmid-transfected HT-29 cells compared to pcDNA plasmid-transfected HT-29 cells (Fig. 5C). These results suggest that the PI3K/Akt signaling pathway is necessary for EGF-induced HO-1 expression. Ser473 residue phosphorylation of Akt by a PI3K-dependent signaling pathway causes enzymatic activation [32]. To confirm the important role of PI3K/Akt in HO-1 expression, we determined Akt Ser473 phosphorylation in response to EGF. As shown in Figure 5D, treatment of HT-29 cells with 10 ng/ml EGF resulted in time-dependent phosphorylation of Akt Ser473. Akt Ser473 phosphorylation peaked at 5∼10 min, and then had declined by 20 min after EGF treatment (Fig. 5D, upper panel). Protein levels of Akt1/2 were not affected by EGF treatment (Fig. 5D, bottom panel).

### c-Src, NADPH oxidase, and PI3K mediate EGF-induced Akt phosphorylation at Ser473 in HT-29 cells

**Figure 6:**
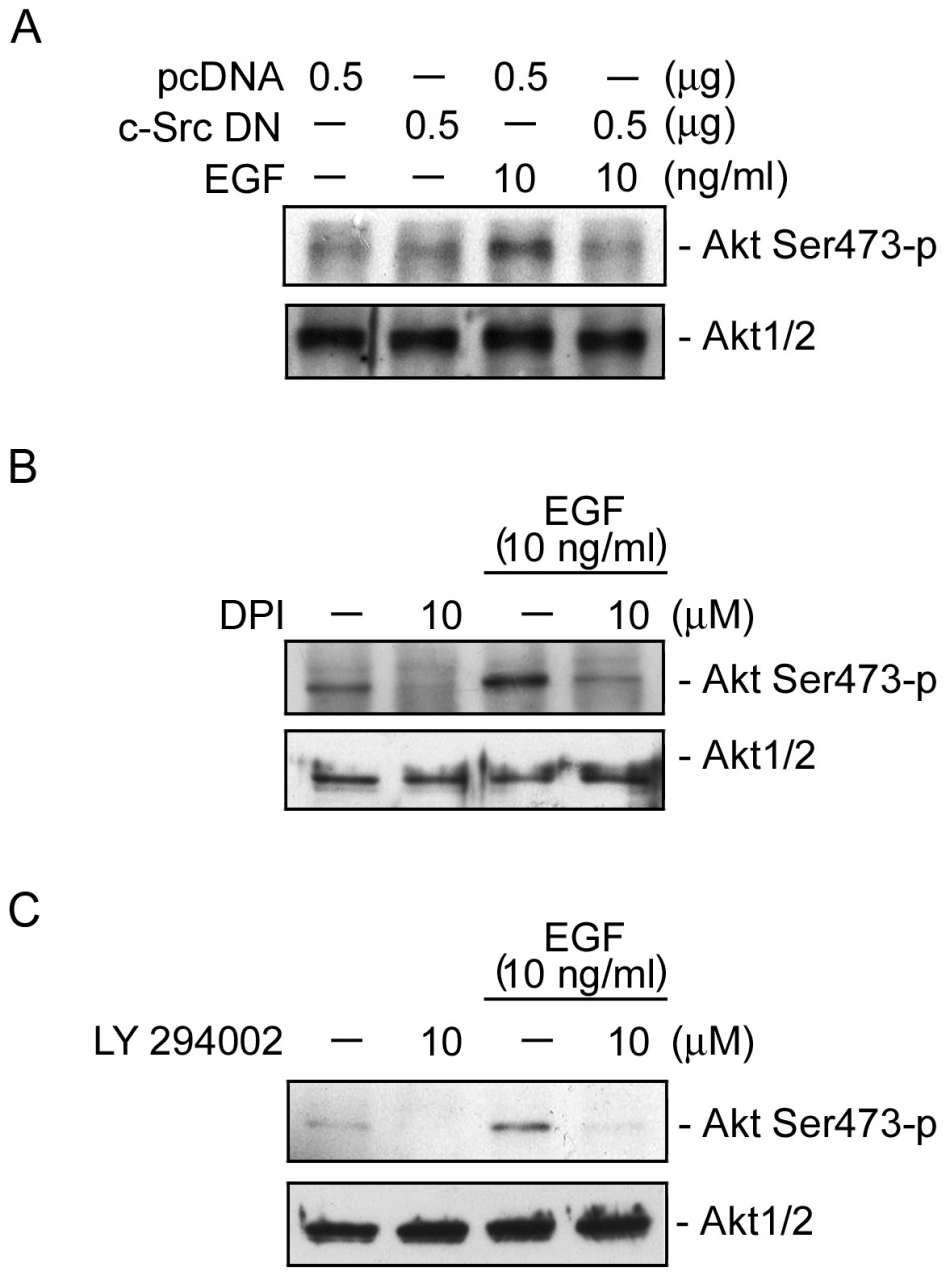
Involvement of c-Src, NADPH oxidase, and PI3K in epidermal growth factor (EGF)-induced Akt Ser473 phosphorylation in HT-29 cells. HT-29 cells were transiently transfected with 0.5 µg of pcDNA or 0.5 µg of a dominant negative mutant of c-Src (c-Src DN) (A) for 24 h, or cells were pretreated for 30 min with 10 µM DPI (B) or 10 µM LY 294002 (C) and then stimulated with 10 ng/ml EGF. After 20 min of stimulation, Akt Ser473 phosphorylation was determined. Immunoblots are representative of three experiments with similar results.

Next, we investigated the roles of c-Src, NADPH oxidase, and PI3K in EGF-induced Akt Ser473 phosphorylation. As shown in Fig. 6, transfection of HT-29 cells with a c-Src DN (0.5 µg) attenuated EGF-induced Akt Ser473 phosphorylation (Fig. 6A). We further examined whether NADPH oxidase and PI3K mediate Akt phosphorylation. We found that EGF-induced Akt Ser473 phosphorylation was also inhibited by DPI (10 µM) (Fig. 6B). Similarly, EGF-induced Akt Ser473 phosphohrylation was also inhibited by LY294002 (10 µM) (Fig. 6C). These results suggest that activation of c-Src, NADPH oxidase, and PI3K occurs upstream of Akt in the EGF-induced signaling pathway.

### NF-κB is involved in EGF-induced HO-1 expression in HT-29 cells

**Figure 7:**
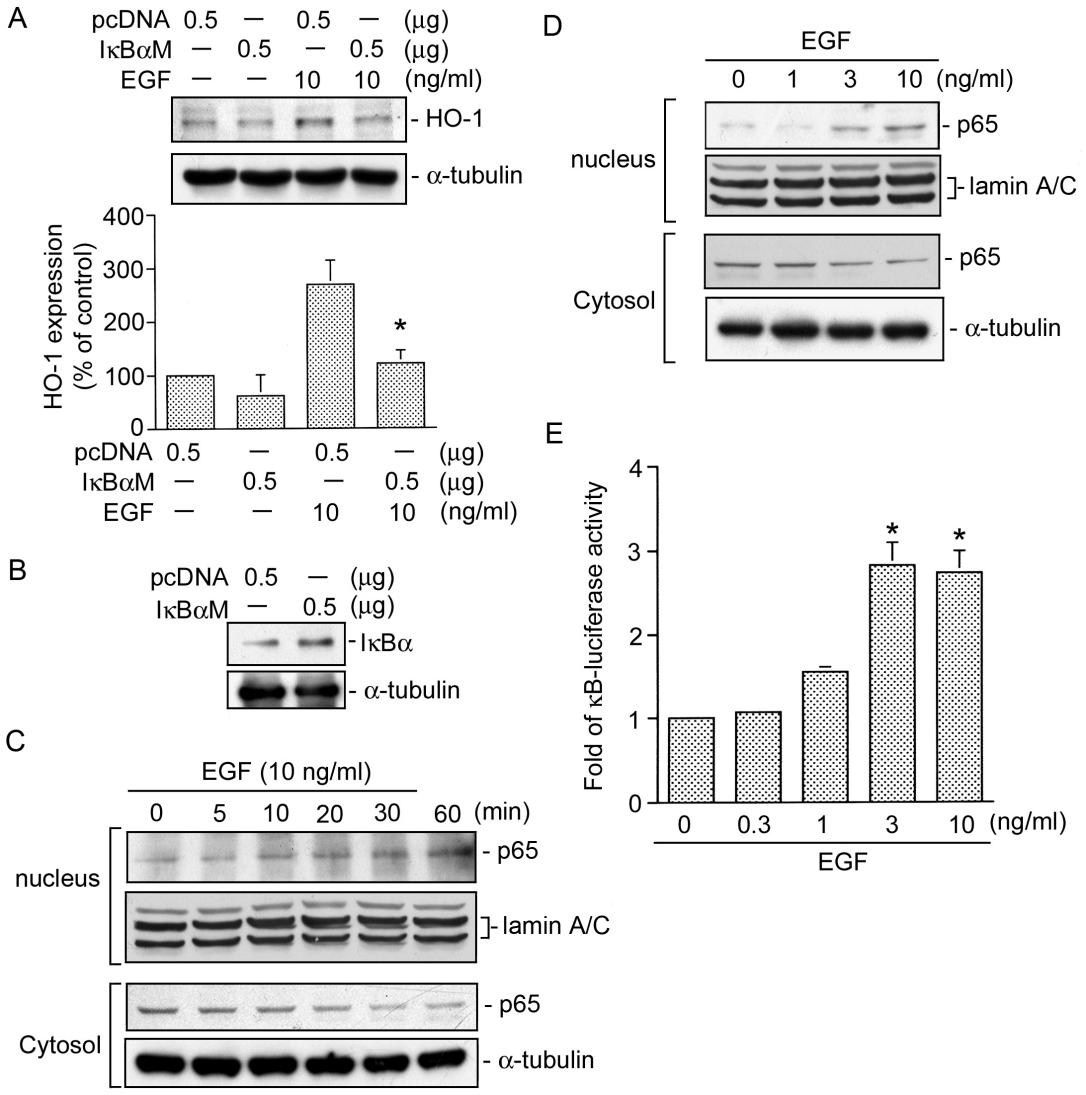
Nuclear factor (NF)-κB is involved in epidermal growth factor (EGF)-induced heme oxygenase (HO)-1 expression in HT-29 cells. A, HT-29 cells were transfected with 0.5 µg pcDNA or 0.5 µg IκBαM for 24 h, and then stimulated with 10 ng/ml EGF for another 18 h. After incubation, HO-1 and α-tubulin protein levels were determined. Immunoblots are representative of three experiments, which are presented as the mean ± SEM. *p<0.05, compared to EGF treatment. B, Cells were transiently transfected with either 0.5 µg of pcDNA or 0.5 µg of IκBαM for 24 h. Levels of IκBα and α-tubulin protein expression were determined by a Western blot analysis. Traces represent results from three independent experiments. HT-29 cells were treated with 10 ng/ml EGF for the indicated time intervals (C) or treated with EGF (1∼10 ng/ml) for 20 min (D). The cytosolic and nuclear fractions were then isolated, and the protein levels of p65 in the cytosolic and nuclear fractions were determined by Western blot analysis. Typical traces represent three experiments with similar results. Lamin A/C and α-tubulin were respectively used as nuclear and cytosolic internal controls. E, HT-29 cells were transiently transfected with 0.5 µg of pGL2-ELAM-Luc and 0.5 µg of pBK-CMV-Lac Z for 24 h. Cells were then stimulated with HT-29 (0.3∼10 ng/ml) for another 24 h. Luciferase activities were determined as described in “Materials and Methods”. The level of induction of luciferase activity was compared to that of cells without EGF treatment. Data are presented as the mean ± SEM. of three experiments performed in duplicate. *p<0.05, compared to the control without EGF treatment.

As previously mentioned, NF-κB activation is necessary for HO-1 expression [33]. To test whether NF-κB activation is involved in EGF-induced HO-1 expression, an IκBαM (an NF-κB inhibitor) was used. Figure 7A shows that transfection of HT-29 cells with 0.5 µg of IκBαM inhibited EGF-induced HO-1 expression by 65±14% (Fig. 7A). Moreover, the IκBα protein was highly expressed in IκBαM plasmid-transfected HT-29 cells compared to pcDNA plasmid-transfected HT-29 cells (Fig. 7B). NF-κB activation was evaluated by the translocation of p65 from the cytosol to nuclei. Treatment of HT-29 cells with 10 ng/ml EGF resulted in marked translocation of p65 from the cytosol to nuclei in a time-dependent manner, with a maximal effect after 30∼60 min of treatment (Fig. 7C). Moreover, we found that treatment of HT-29 cells with EGF (1∼10 ng/ml) produced concentration-dependent increases in p65 translocation from the cytosol to the nuclei (Fig. 7D). Next, to directly determine NF-κB activation after EGF treatment, HT-29 cells were transiently transfected with pGL2-ELAM-κB-luciferase as an indicator of NF-κB activation. As shown in Figure 7E, EGF (0.3∼10 ng/ml) treatment of HT-29 cells for 24 h caused a concentration-dependent increase in κB-luciferase activity. Cells treated with 10 ng/ml EGF showed an increase in κB-luciferase activity of 2.7±0.3-fold (*n = *3) (Fig. 7E). These results suggested that NF-κB activation is important for EGF-induced HO-1 expression in HT-29 cells.

### c-Src, NADPH oxidase, PI3K, and Akt mediates EGF-induced κB-luciferase activity

**Figure 8:**
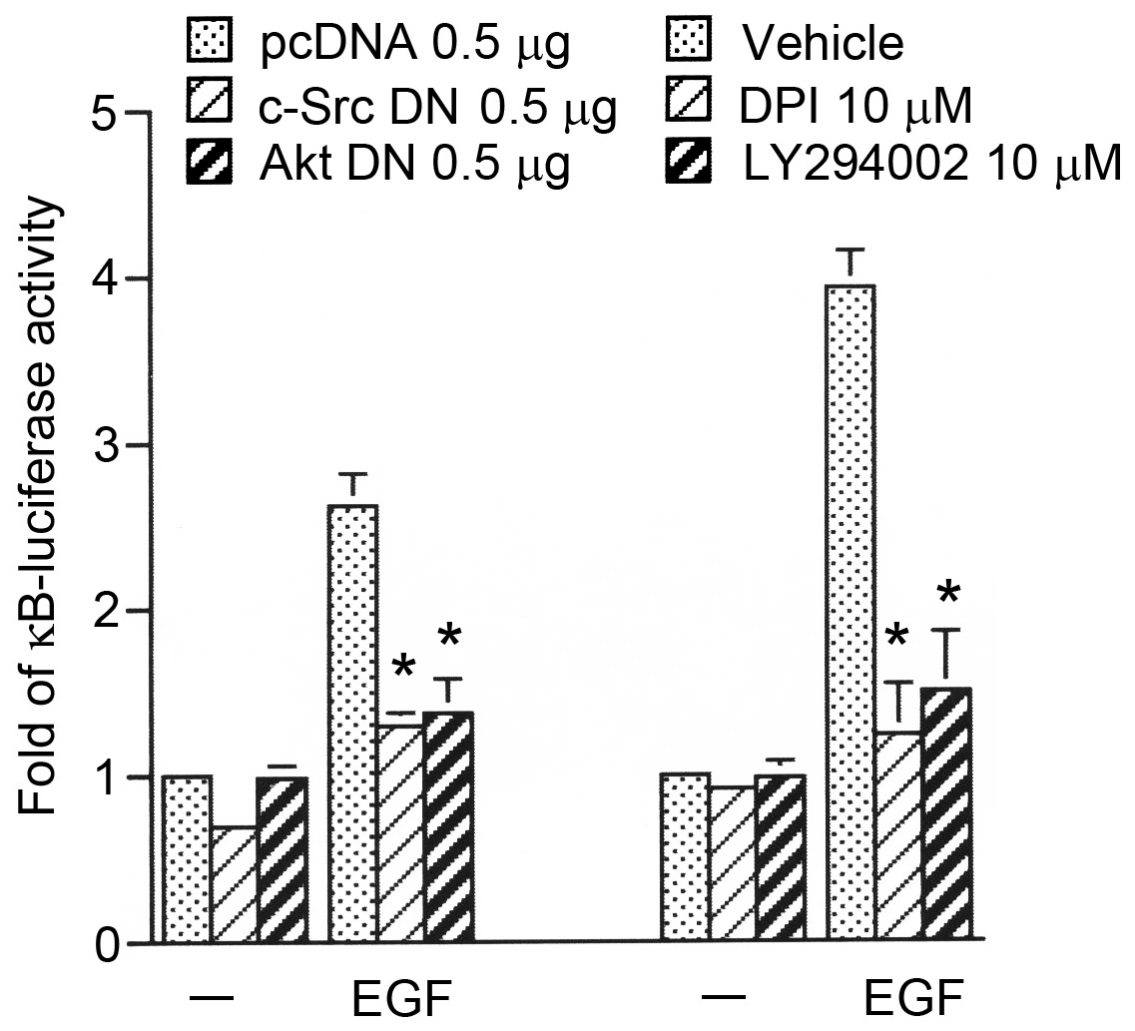
Involvement of c-Src, NADPH oxidase, PI3K, and Akt in epidermal growth factor (EGF)-induced increases in κB-luciferase activity in HT-29 cells. HT-29 cells were transiently transfected with 0.5 µg of pGL2-ELAM-Luc and 0.5 µg of pBK-CMV-Lac Z for 24 h and were either cotransfected with pcDNA (0.5 µg), c-Src DN (0.5 µg), and Akt DN (0.5 µg), or cells were pretreated with LY 294002 (10 µM) or DPI (10 µM) for 30 min, before incubation with 10 ng/ml EGF for 24 h. Cells were then harvested for the κB-luciferase assay as described in Fig. 7E. Data are presented as the mean ± SEM of three experiments performed in duplicate. *p<0.05, compared to EGF treatment.

To further explore whether EGF-induced NF-κB activation occur through the c-Src, NADPH oxidase, PI3K, and Akt pathways, HT-29 cells were treated with a c-Src DN (0.5 µg), DPI (10 µM), LY 294002 (10 µM), and an Akt DN (0.5 µg), which respectively inhibited the EGF-induced increase in κB-luciferase activity by 51±1%, 89±10%, 82±10%, and 76±13% (*n = *3) (Fig. 8). Taken together, these results suggest that activation of c-Src, NADPH oxidase, PI3K, and Akt is required for EGF-induced NF-κB activation in HT-29 human colon cancer cells.

### Involvement of HO-1 in EGF-induced colon cancer cell proliferation

**Figure 9:**
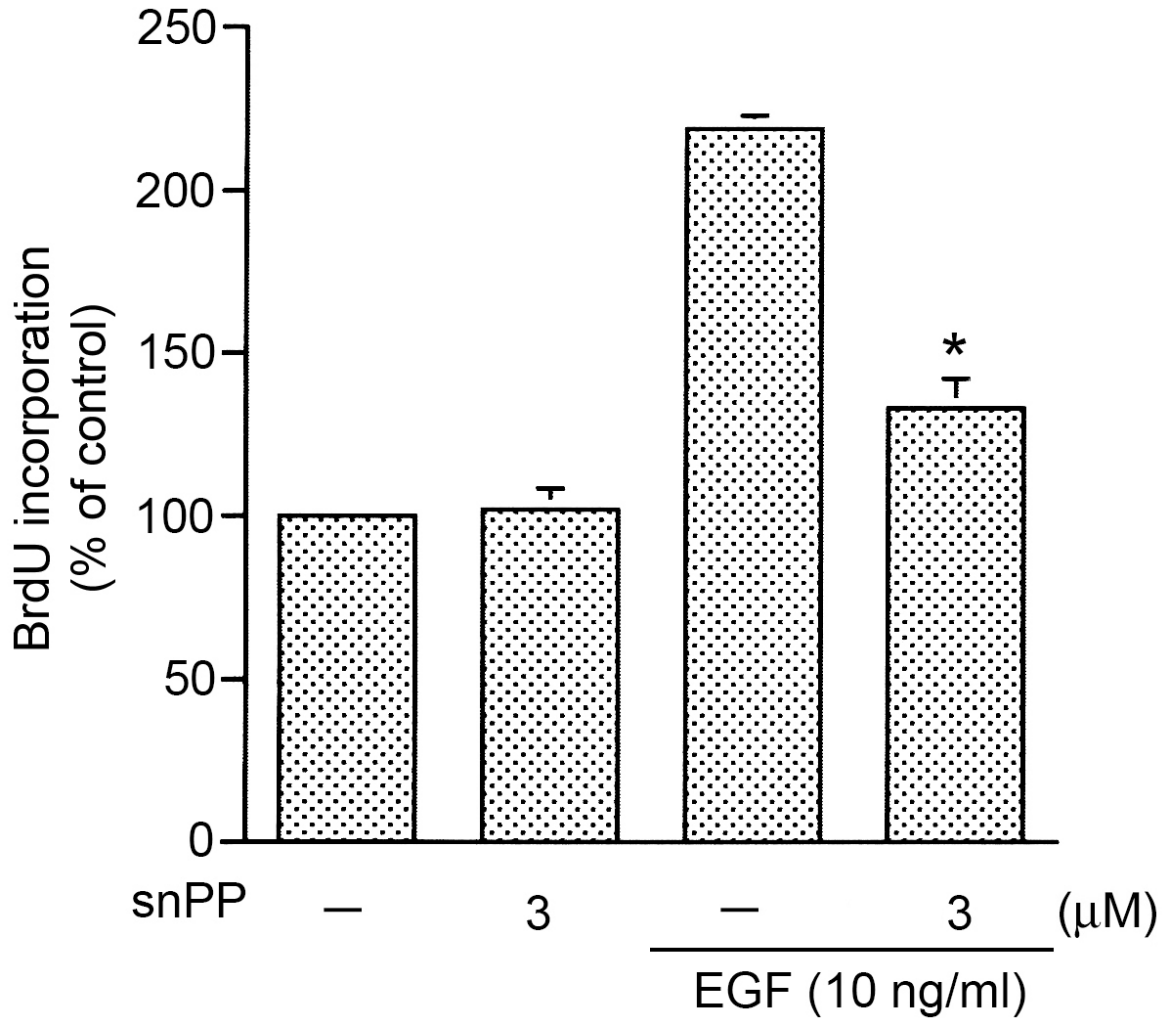
Involvement of heme oxygenase (HO)-1 in epidermal growth factor (EGF)-induced proliferation of HT-29 cells. Cells were pretreated for 30 min with 3 µM Sn(IV)protoporphyrin-1X (snPP) and then stimulated with 10 ng/ml EGF for another 48 h. A BrdU cell proliferation assay was carried out as described in “Materials and Methods”. Data are presented as the mean ± SEM of three experiments. *p<0.05, compared to EGF treatment.

A previous report demonstrated that EGF induced colon cancer proliferation [34]. Another study indicated that HO-1 plays a protective role in cancer cells [5]. Next, to examine whether HO-1 mediates EGF-induced colon cancer cell proliferation, a HO-1 inhibitor (snPP) was used. As shown in Fig. 9, we found that snPP (3 µM) significantly inhibited 10 ng/ml EGF-induced cell proliferation by 74±8% (*n* = 3) (Fig. 9). This result indicates that induction of HO-1 expression contributes to EGF-induced cell proliferation in HT-29 cells.

## Discussion

A growing body of evidence has demonstrated that EGF mediates a variety of cellular events, including cancer cell progression and metastasis. Additionally, EGF induces protein expression involved in cell cycle progression, such as membrane-type 1 metalloproteinase and HO-1 in colonic epithelial cells [35], [36]. HO-1 plays critical roles in the processes of anti-inflammation, tissue protection, and anti-oxidative stress reaction. It was reported that HO-1 can promote tumorigenesis, cell proliferation, and metastasis of many tumors. In the present study, we investigated the effects of EGF-induced HO-1 expression in human HT-29 colon cancer cells. Our data demonstrated that EGF stimulated activation of c-Src, NADPH oxidase, PI3K, and Akt, which in turn induced NF-κB activation and HO-1 expression in HT-29 cells. Moreover, HO-1 mediates EGF-induced colon cancer cell proliferation.

Several studies indicated that the induction of HO-1 in response to various stimuli is a consequence of mRNA and protein syntheses in human cancer cells [37]. The *HO-1* gene processes multiple potential regulatory transcription factor-binding sites, including HSE, NF-κB, AP-2, and interleukin-6-responsive elements [14], [15], suggesting a potential role for these transcription factors in modulating HO-1 induction. Several studies suggested that NF-κB plays an important role in regulating cancer cell HO-1 expression. In human malignant human oral keratinocyte cells, nicotine-induced HO-1 expression requires NF-κB activation [18]. Our previous study indicated that TGF-β-induced HO-1 expression depends on the NF-κB signaling pathway in human lung adenocarcinomas [6]. Results of the present study showed that NF-κB activation is essential for HO-1 expression stimulated by EGF in human colon cancer cells. This is based on the fact that IκBαM (an NF-κB inhibitor) inhibited EGF-induced HO-1 expression. Furthermore, EGF induced increases in p65 translocation from the cytosol to nuclei and κB-luciferase activity. Consistent with our study, NF-κB was found to play a critical role in regulating EGF-dependent increases in *HO-1* gene expression in A549 cells [38]. Taken together, these results suggest that NF-κB activation is necessary for HO-1 expression by EGF in human colon cancer cells.

The PI3K/Akt signaling pathway was demonstrated to play important roles in promoting cell proliferation and survival [39]. PI3K/Akt was shown to be required for activation of transcription factors, including NF-κB, and mediates gene expression in human colon cancer cells by various stimuli [40]. Tsai *et* *al.* (2012) reported that in human non-small cell lung cancer cells, high expression of HO-1 was inhibited by PI3K and Akt inhibitors [41]. Dal-Cim *et* *al.* (2012) showed that in human neuroblastomas, LY 294002 (a PI3K inhibitor) inhibited guanosine-mediated HO-1 expression [42]. In the current study, we present data to support the role of the PI3K/Akt signaling pathway in EGF-induced HO-1 expression in human colon cancer cells. We found that LY 294002 and the Akt DN both significantly inhibited EGF-induced HO-1 expression. Moreover, we found that EGF induced an increase in Akt Ser473 phosphorylation, which was inhibited by LY 294002. In addition, we further examined the PI3K/Akt signaling pathway, a major cascade mediating activation of NF-κB signal transduction in human colon cancer cells [43]. A previous study indicated that PI3K/Akt-mediated induction of NF-κB transcriptional activity is necessary and sufficient for MUC2 expression [43]. Our previous report also suggested that PI3K/Akt-dependent NF-κB activation is necessary for TGF-β-induced HO-1 expression in human lung epithelial cells [6]. In this study, we found that the EGF-induced increase in κB-luciferase activity was attenuated by LY 294002 and an Akt DN, indicating that the PI3K/Akt pathway is involved in EGF-induced NF-κB activation. Several reports demonstrated that NADPH oxidase was required for activation of the Akt signaling pathway and mediate protein expression by various stimuli [44], [45]. Feliers *et* *al.* (2006) reported that DPI (a NADPH oxidase inhibitor) completely abolished angiotensin II-induced Akt phosphorylation in proximal tubular epithelial cells [44]. Another study demonstrated that DPI also completely inhibited vascular endothelial growth factor-induced Akt phosphorylation in endothelial cells [45]. In this study, we found that DPI almost completely attenuated EGF-induced Akt phosphorylation at Ser473 in HT-29 cells. This result suggests that EGF-induced Akt phosphorylation is dependent on NADPH oxidase.

Activated c-Src plays vital roles in several cellular events, including cell survival, proliferation, migration, cancer formation, and inflammatory gene expression [46]. A previous report showed that c-Src is a downstream molecule of the EGFR, and we raised the question of whether c-Src plays an important role in EGF-induced HO-1 expression. In human conjunctival goblet cells, EGF-induced cell proliferation requires c-Src activation [12]. c-Src is involved in thrombin-induced HO-1 expression in human synovial fibroblasts [20]. A previous report by Brunton *et* *al.* showed that EGF-induced c-Src activation is required for progression of human colon cancer cells [47]. In this study, we found that a c-Src DN inhibited EGF-induced HO-1 protein expression. Moreover, EGF induced an increase in c-Src phosphorylation at Tyr416 in human colon cancer cells. These results indicate that c-Src plays a crucial role in EGF-induced HO-1 expression. NADPH oxidase was been reported to play a critically essential role in agent-mediated ROS generation, cancer survival, and tumorigenesis [48]. Recently, a large of body of evidence implied that NADPH oxidase generates ROS involvement in HO-1 expression by various stimuli in different cells types [29], [49]. He *et* *al.* reported that NADPH oxidase mediates hyperglycemia-induced HO-1 expression in retinas of diabetic mice [49]. Another study demonstrated that cigarette smoke extract induced HO-1 expression through NADPH oxidase and ROS in cerebral endothelial cells [29]. In this study, we further demonstrated the roles of NADPH oxidase and ROS in EGF-induced HO-1 expression in human colon cancer cells. We found that DPI (an NADPH oxidase inhibitor) and glutathione (an ROS inhibitor) both inhibited EGF-induced HO-1 expression. Moreover, we found that EGF induced the translocation of p47*^phox^*, a regulator of NADPH oxidase, from the cytosol to the membranes. Furthermore, EGF-induced ROS production was inhibited by DPI. Therefore, the results suggest that NADPH oxidase-dependent ROS production is involved in EGF-induced HO-1 expression in HT-29 cells.

Our previous study indicated that thrombin-induced NF-κB activation is dependent on c-Src activity in human lung epithelial cells [25]. Brady *et* *al.* (2011) reported that inhibition of c-Src leads to a reduction in NF-κB activation in colorectal cancer cells [50]. Moreover, several studies demonstrated that NF-κB activation by advanced oxidation protein products and IL-1β depends on NADPH oxidase activation in fibroblast-like synoviocytes cells and human intestinal epithelial cells [51], [52]. In this study, we present data that confirmed the role of c-Src and NADPH oxidase in EGF-induced NF-κB activation in human colon cancer cells. We found that a c-Src DN and DPI both inhibited EGF-induced κB-luciferase activity. These results suggest that c-Src and NADPH oxidase are upstream molecules of NF-κB after EGF stimulation of human colon cancer cells.

Several studies demonstrated that EGF plays an important role in the development of colon cancer [11]. Moreover, overexpression of HO-1 plays a protective role in cancer cell survival [9], [10]. A previous report showed that EGF can induce cancer cell proliferation [34]. In this study, we found that the EGF induced an increase in colon cancer cell proliferation, which was inhibited by snPP (an HO-1 inhibitor). This result suggests that HO-1 is involved in EGF-induced colon cancer cell proliferation.

**Figure 10:**
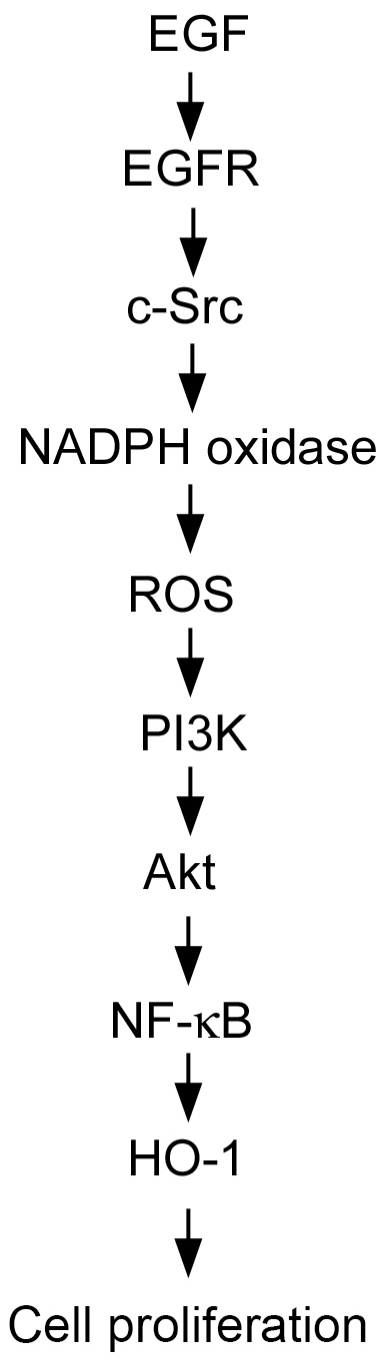
Schematic summary indicating how signal transduction by epidermal growth factor (EGF) induces HO-1 expression in human HT-29 colon cancer cells. EGF activates the c-Src, NADPH oxidase, and PI3K/Akt pathways, which in turn induces NF-κB activation and HO-1 expression in human colon cancer cells. Moreover, HO-1 is involved in EGF-induced colon cancer cell proliferation.

In conclusion, this study provides fundamental information on the regulatory molecular mechanisms of EGF-induced HO-1 expression through the c-Src, NADPH oxidase, ROS, PI3K, and Akt signaling pathway in increasing NF-κB activation and HO-1 protein expression in human HT-29 colon cancer cells. Moreover, induction of HO-1 expression mediates EGF-induced colon cancer cell proliferation. Figure 10 is a schematic representation of the signaling pathway involved in the enhancement of HO-1 expression in response to EGF in human colon cancer cells. Our results provide a mechanism linking EGF and HO-1, and they support the development of therapeutic strategies to reduce colon cancer progression caused by EGF.
